# Current knowledge of Huntington's disease-like 2 genetic testing, clinical presentation, and patient experiences: A systematic review

**DOI:** 10.1177/18796397251411109

**Published:** 2026-01-21

**Authors:** Katharina Hoffmann, Stephanie White, Adrienne Sexton

**Affiliations:** 1 Graduate School of Health, 550179 University of Technology Sydney, Sydney, Ultimo NSW, Australia; 2 Genomic Medicine & Parkville Familial Cancer Centre, 90134 The Royal Melbourne Hospital and Peter MacCallum Cancer Institute, Parkville, VIC, Australia; 3 Department of Medicine-RMH, The University of Melbourne, Parkville, VIC, Australia; 4 Genetic Counselling Service, Epworth Freemasons, East Melbourne VIC, Australia

**Keywords:** Huntington disease-like 2, chorea, cognition disorders, dementia, neurodegenerative disorders, genetic counselling, genetic testing, diagnosis

## Abstract

**Background:**

Huntington's Disease-like 2 (HDL2) presents complexities in diagnosis due to its similarity to Huntington's Disease (HD). Limited research highlights gaps in knowledge about management and genetic counselling for the condition. HDL2 is rare but an important differential diagnosis for individuals with HD-like symptoms who have tested negative for HD.

**Objective:**

This review aimed to synthesise published clinical and genetic data on HDL2, identify knowledge gaps, and serve as a resource for healthcare professionals supporting individuals affected by or at risk of HDL2.

**Methods:**

A mixed method integrative systematic review of four databases (Medline, Embase, Scopus, and PsycINFO) generated 323 peer-reviewed articles, of which 36 were included. Data about clinical features, genetic testing and counselling, and patient experiences were interpreted via narrative synthesis.

**Results:**

The majority of included studies explored the clinical features, genetic testing results and medical histories of individuals with HDL2. A total of 109 people with HDL2 were reported. Limited data was obtained about genetic counselling, management and support, and experiences of those with HDL2 and their families. Findings related to seven categories: DNA repeat length and impact on phenotype, age of onset and disease duration, family history, African ancestry, neurological characteristics, clinical characteristics, and experiences and support.

**Conclusions:**

This review highlights the importance of understanding the reduced penetrance range and early psychiatric symptoms in HDL2 for accurate genetic counselling and interpretation of test results. Adapting existing protocols for HD and qualitatively collecting patient experiences can inform the development of a HDL2 genetic testing and counselling protocol.

## Plain language summary

Huntington's Disease-like 2 (HDL2) is a rare condition that can look very similar to Huntington's Disease (HD), which often makes it hard to diagnose. People who have symptoms like those seen in HD but test negative for HD may have HDL2 instead. This paper adds a new depth compared to a previous overview on HDL2 by Krause and co-authors in 2024.^1^ Here, we have used systematic methods to look at all the available research on HDL2 to better understand the condition and to help doctors and genetic counsellors support individuals and families affected by it. We searched four major research databases and found 36 relevant studies including 109 people with HDL2. Most of these focused on the medical and genetic features of people with HDL2. However, we found very little research on how to support people living with the condition or how to provide genetic counselling to families at risk. The main findings included information about how the condition is inherited, the role of African ancestry, symptoms that often appear early (especially psychiatric symptoms), and the length of time people have the condition for. Our review shows that more research is needed on how to support people with HDL2 and their families. Understanding early symptoms involved can help improve diagnosis and counselling. In the meantime, adapting support approaches used for Huntington's Disease may be helpful.

## Introduction

Genetic health care encompasses a multidisciplinary approach that involves various health professionals.^2^ This collaborative process can support individuals to understand the genetic factors behind genetic conditions and the resulting medical, psychological, and familial implications.^2^ Health professionals such as clinical geneticists, genetic counsellors, neurologists, neuropsychiatrists, psychologists, and primary care physicians work together to interpret family and medical histories, assess the likelihood of disease, facilitate family communication, and provide management and support.^2^ Conditions like Huntington's disease (HD; Phenotype MIM number 143100) exemplify the complexities involved in genetic counselling due to the significant medical, psychological, social and familial impacts.^3^ When genetic testing for HD yields negative results, there is a need to be aware of differential diagnoses including other rare genetic conditions with similar movement, psychiatric and behavioural features.^4^ One such possibility is Huntington's disease-like 2 (HDL2; Phenotype MIM number 606438).^4^ This review aims to inform genetic health care for people with a potential or confirmed diagnosis of HDL2.

HDL2 is an adult-onset, autosomal dominant neurodegenerative disorder that affects the brain, resulting in a range of physical, cognitive and neuropsychiatric symptoms such as chorea, dementia, depression and irritability.^5,6^ While HD is caused by an expansion of CAG repeats in the *HTT* gene (MIM number 613004) on chromosome 4, HDL2 results from similar repeat expansions in the *JPH3* gene (MIM number 605268) on chromosome 16q24.^5,6^ The *JPH3* gene encodes the junctophilin-3 protein, which is predominantly expressed in the brain and therefore is hypothesised to be important for forming a junctional complex.^6,7^ This complex appears to facilitate the release of calcium ions, aiding in cell signal transmission.^6^ Thus, junctophilin- 3 is believed to play a crucial role in signalling both within and between nerve cells in the brain.^8^

HDL2 is reported to be nearly clinically and pathologically indistinguishable from HD.^9,10^ Previous reviews, notably by Krause et al. (2024), have summarised the medical and genetic data on this rare condition.^1^ While HDL2 shares many clinical similarities with other neurodegenerative conditions such as HD and chorea-acanthocytosis, a study reviewing 73 HDL2 cases concluded that individuals with HDL2 almost always have confirmed or probable African ancestry.^10,11^ Since its discovery, there have been no cases of HDL2 reported in individuals of solely European or Asian heritage.^10,12^ Differentiating between these conditions with overlapping phenotypes, including movement abnormalities, cognitive decline, and psychiatric symptoms, is difficult.^13^ Hence, the ability to recognise when HDL2 is a possibility will allow health professionals such as neurologists, clinical geneticists, neuropsychiatrists and genetic counsellors to choose the appropriate genetic tests, efficiently and accurately confirm the diagnosis and provide tailored management strategies.^13^

Understanding the patient experience of living with HDL2 and receiving genetic counselling can help genetic counsellors and other health professionals to offer holistic care, addressing clinical, psychosocial, emotional, and practical concerns.^14^ The existing literature provides very limited insight into these experiences.^1,15^ To date, reviews on HDL2 have not systematically examined patient perspectives towards support needs or experiences of genetic counselling.^1,15^ Drawing on insights from HD literature, it can be inferred that high-quality genetic counselling is equally crucial for HDL2 for affected patients and their unaffected, at-risk family members.^16,17^ For those undergoing presymptomatic testing for HD, it is essential to provide comprehensive and patient-centred information about the testing protocol as well as adequate support, particularly in the pre-test period.^16,17^ Family members caring for HD patients often face unexpected changes in family roles and dynamics.^18^ As symptom onset is typically in mid-adulthood and people with these conditions experience a range of progressive symptoms affecting personal relationships, executive function and ability to work, caregiving for someone with HD can be exceptionally challenging and stressful, with caregivers potentially being children or spouses of any age.^18,19^ Hence, it is evident that emotional and psychological impact of such testing cannot be underestimated. Therefore, it is important to understand what evidence is available regarding support and counselling needs for individuals undergoing testing for HDL2 and their family members and caregivers.^16–19^

For these reasons, a systematic review focusing on HDL2 clinical presentation, genetic testing and patient experiences is important in synthesising current knowledge and identifying gaps in the literature and research. The review questions are:

1. What information about HDL2 is currently available and can be used to enhance the process of genetic testing, diagnosis, and genetic counselling for HDL2?
2. What information about patient and family members experiences is currently available to enhance our understanding of what patients with HDL2 require for additional support?

## Materials and methods

A mixed methods integrative systematic review was chosen for its ability to encompass various research paradigms relevant to HDL2 research.^20^ Numerical data, such as *JPH3* repeat expansion size and age of onset, aligns with the positivist paradigm, while qualitative data exploring opinions and perspectives adds depth learnt from the constructivist paradigm.^20,21^ By incorporating both paradigms, this methodologically inclusive approach broadened the accessibility and utility to a larger range of readers including genetic counsellors and other clinical decision-makers.^22^

### Search strategy

This systematic search strategy was developed in consultation with an information services librarian and aimed to locate peer-reviewed, published studies pertinent to the research topic. Key words included HDL2, genetic testing, genetic counselling, diagnosis, patients, and family. Each key word was expanded to account for variations in spelling, acronyms and regional differences. Terms were cross-referenced with the National Library of Medicine's MeSH terms (Medical Subject Headings). The keywords and MeSH terms were combined with Boolean operators in PubMed, PsycINFO, Embase and Scopus (Supplementary Table 1.1).

### Eligibility criteria

The eligibility criteria were formulated based on the PICo framework (Population, Phenomenon of Interest, and Context).^23^ This framework aligns with the research questions by enabling the exploration of various phenomena of interest which include genetic testing, genetic counselling, diagnosis, and the lived experiences of individuals affected by HDL2 or involved in caregiving for someone with HDL2. We included articles published between 2001 and 2024 as the *JPH3* gene and HDL2 were initially documented in 2001.^7^ Articles were also included if they were published in English in a peer-reviewed journal. Eligibility criteria are provided in Table 1.

**Table 1.** Inclusion and exclusion criteria, developed in accordance with the PICo^a^ criteria.

	Include	Exclude
**Study Characteristics**	Articles published after 2001 Articles published in English Articles in peer-reviewed journals Articles using qualitative, quantitative, mixed method studies or case reports	Articles published before 2001 Articles published in another language other than English Articles not in peer-reviewed journals Review articles Conference abstracts Animal studies, somatic studies, in vitro studies, studies which did not report on HDL2
**Population**	Individuals with a HDL2 diagnosis	Studies in animal or other models Individuals without a HDL2 diagnosis
**Phenomena of Interest**	Diagnostic testing for HDL2 Variant detection rate Pre-symptomatic testing for HDL2 Pre-natal testing for HDL2 Diagnosis process for HDL2 Allele sizes, ancestry, clinical features, family history, clinical evaluations, and neuroimaging Genetic counselling for HDL2 Pre-test period, genetic counselling appointments, follow-up appointments Communication between family members Management of HDL2 Disease progression Areas of strength Areas of weakness (and targeted interventions, if any) Lived experience of HDL2 patients and their family members Psychological impact of HDL2 for patients and family members	Studies reporting solely on diseases other than HDL2 (e.g., Huntington's disease, other neurodegenerative disorders, other Huntington's disease phenocopies).
**Context**	Studies from any country or location Studies conducted in a clinical setting	Studies not conducted in a clinical setting

^a^ Population: Specifies the characteristics of the individuals included in the studies, Context: Describes the settings and circumstances in which the studies were conducted, Phenomena of interest: specific topics, or experiences that are being studied

### Screening and selection

**Figure 1.**
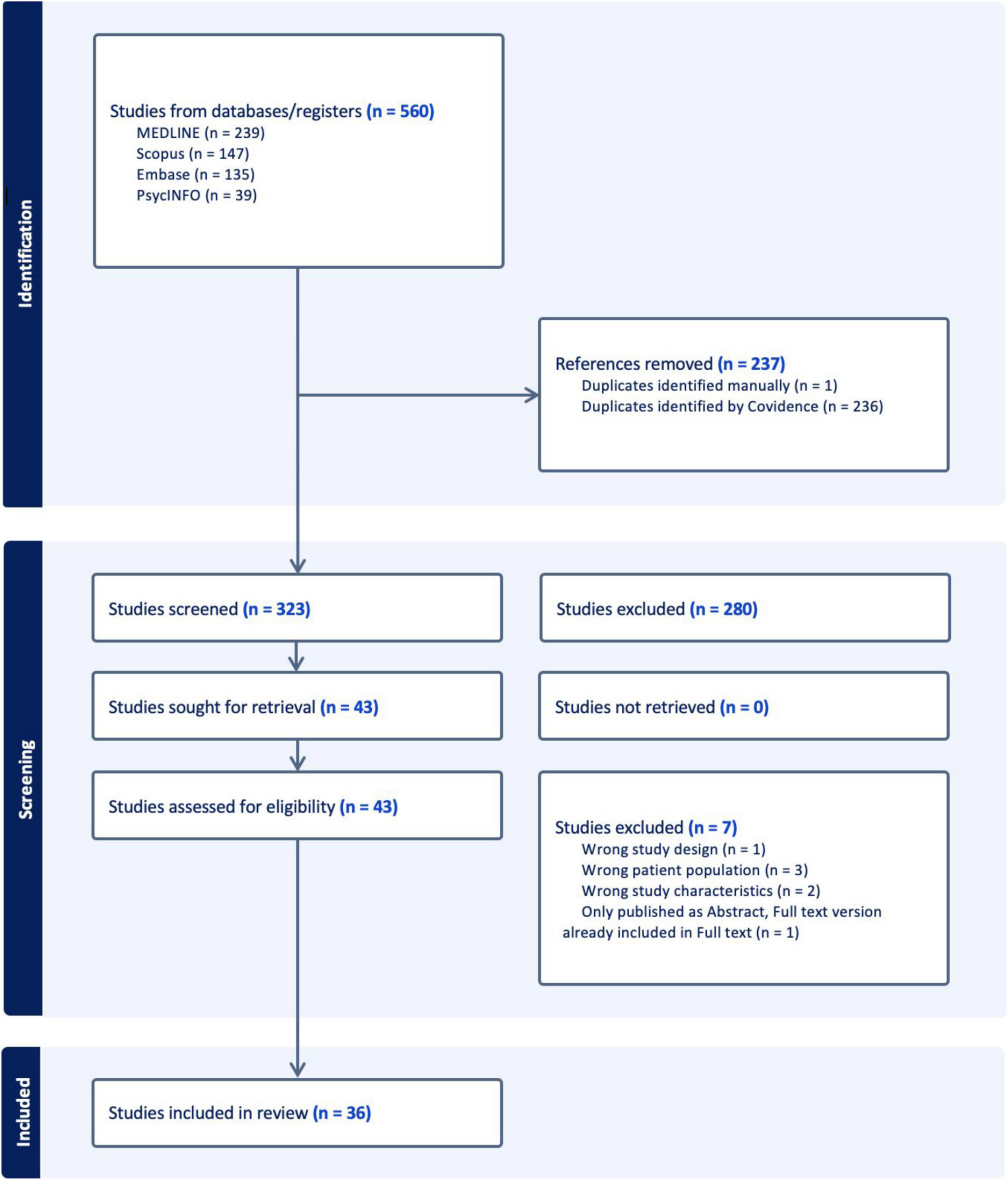
PRISMA flow diagram summarising the systematic selection process. Following duplication, 323 unique articles were screened against eligibility criteria, resulting in 36 articles for inclusion.

The first stage required selection and screening to be based on the title and abstract of the article only, while the second stage required an examination of the full text and was performed using Covidence software.^24,25^ Two reviewers independently screened 10% of papers for eligibility at both stages, and a Cohen's kappa was calculated to assess interrater reliability.^26^ Any discrepancies were then resolved through discussion.^24^ If discussion failed to resolve the discrepancy, the assistance of a third researcher was sought.^24^ A predetermined hierarchy of exclusion criteria was agreed upon to ensure consistency and to streamline the screening process by prioritising the most relevant exclusion reasons. Following three rounds of co-screening, Cohen's kappa reached 1, signifying a 100% agreement rate and meeting our predetermined threshold of 0.81.^26^ Subsequently, one reviewer (K.H.) proceeded to independently screen the remaining papers.^24,25^ For any text excluded during the full-text screening stage, a justification was recorded as summarised in the PRISMA flow diagram (Figure 1).^27^

### Data extraction

Data were extracted based on three categories including general data, individual study characteristics and main findings. General data included the author, title, source and year of publication. Individual study characteristics included type of study, methods, limitations, aims, number and characteristics of participants. The main findings were split into subcategories including phenomena of interest, key findings, recommendations from study, future research recommendations and authors’ conclusions.

### Risk of bias

Quality assessments were conducted using JBI Critical Appraisal Tools, which are tailored to different study designs.^28–30^ These tools assess the quality of observational studies, including case reports, case series, case-control studies, and cross-sectional studies and were chosen because all papers identified were observational in nature.^28–30^ In the absence of experimental designs focusing on exposures and outcomes, these tools ensure a comprehensive summary of the best available evidence beyond randomised controlled trials.^28–30^ The first 10% of the critical appraisal was conducted independently by two researchers.^31^ Discrepancies were resolved through discussion.^30^ In case of disagreements, a third investigator was consulted to reach a final decision.^30^ We identified, acknowledged, and carefully considered studies with higher risk scores in our narrative synthesis. In Table 2, we defined studies as high risk if they received a score of <50% “Yes”, moderate risk with a score of 50%-69% “Yes”, and low risk with a score of ≥70% “Yes”. Despite their higher risk scores, we opted not to exclude them from the systematic review. Instead, where high-risk studies presented outlying or inconsistent results, we flagged these in the synthesis to help contextualize their potential influence on overall conclusions. Given the rarity of HDL2, each study will contribute to the comprehensive understanding necessary to capture the full scope and nuances of HDL2.

**Table 2.** Summary of included papers on genetic testing and counselling for Huntington’s Disease Like 2, including country of origin, study design, method, type of data extracted and risk of bias.

Author	Country of origin	Study design	Method	Type of data extracted	Risk of bias
Anderson et al. (2017)^61^	South Africa	Case-Control	Quantitative	CF	Moderate
Anderson et al. (2019)^9^	South Africa	Cross-Sectional	Mixed Methods	CF, MH	Low
Anderson et al. (2019)^46^	South Africa	Cross-Sectional	Quantitative	CF	Low
Baine, Krause and Greenberg (2016)^55^	South Africa	Cross-Sectional	Quantitative	GT	Low
Bardien et al. (2007)^51^	South Africa	Case Series	Quantitative	CF, MH	Moderate
Bauer et al. (2002)^57^	USA	Cross-Sectional	Quantitative	CF, MH, GT	High
Bocoum et al. (2024)^36^	Mali	Case Series	Quantitative	CF, MH, GT, MS, E	Low
Castilhos et al. (2014)^59^	Brazil	Case Series	Quantitative	CF, MH, GT	Low
De Oliveira et al. (2020)^38^	Brazil	Case Report	Qualitative	CF, MH, GT	Moderate
Ferreira-Correia et al. (2020)^43^	South Africa	Case-Control	Quantitative	CF	Low
Ferreira-Correia, Krause and Anderson (2020)^48^	South Africa	Cross-Sectional	Quantitative	CF, MS, E	Low
Ferreira-Correia et al. (2022)^47^	South Africa	Case-Control	Quantitative	CF	Low
Fischer, Licht and Mendez (2012)^14^	USA	Case Report	Qualitative	CF, MH, GT, MS	Moderate
Greenstein et al. (2007)^45^	USA	Case Series	Mixed Methods	CF, MH, GT, E	Moderate
Ibanez et al. (2024)^91^	UK	Cross-Sectional	Quantitative	CF	Low
Krause et al. (2015)^10^	South Africa	Case Series	Quantitative	CF, MH, GT	Moderate
Magazi et al. (2008)^52^	South Africa	Case Series	Qualitative	CF, GT	Low
Margolis et al. (2004)^49^	USA	Case Series	Quantitative	CF, MH, GT	Moderate
Mariani et al. (2016)^92^	France	Case Series	Quantitative	CF, MH, GT	Low
Mulroy et al. (2020)^60^	UK	Case Report	Qualitative	CF, MH, GT	Moderate
Narotam-Jeena et al. (2024)^35^	South Africa	Case Series	Quantitative	CF, MH, GT, E, GC	Moderate
Nguyen et al. (2022)^56^	France	Cross-Sectional	Quantitative	CF, MH, GT	Low
Ocampo, Daimari and Oyekunle (2018)^12^	Botswana	Case Report	Qualitative	CF, GT, MS, GC	Moderate
Paradisi, Ikonomu and Arias (2013)^50^	Venezuela	Case Series	Quantitative	CF, MH, GT	Moderate
Paradisi, Ikonomu and Arias (2024)^34^	Venezuela	Case Series	Quantitative	CF, MH, GT	Moderate
Ramirez-Garcia et al. (2022)^37^	Mexico	Case Series	Quantitative	CF, MH, GT	Low
Rodrigues et al. (2008)^42^	Brazil	Case Series	Mixed Methods	CF, MH, GT, MS	High
Rodrigues et al. (2011)^93^	Brazil	Cross-Sectional	Quantitative	MH, GT	Moderate
Ruscitti et al. (2022)^39^	Italy	Case Report	Quantitative	CF, MH, GT, MS	Low
Santos et al. (2008)^58^	Brazil	Case Report	Quantitative	CF, MH, GT	Low
Sizer et al. (2012)^40^	South Africa	Cross-Sectional	Quantitative	GT, GC	Low
Stevanin et al. (2003)^53^	France	Cross-Sectional	Quantitative	CF, MH, GT	Moderate
Vasconcellos et al. (2017)^41^	Brazil	Case Report	Qualitative	CF, GT, MS	Low
Walker et al. (2003)^44^	USA	Case Series	Mixed Methods	CF, MH, GT, E	High
Walker et al. (2003)^11^	USA	Case Series	Quantitative	CF, GT	High
Wild et al. (2008)^54^	UK	Case Series	Quantitative	CF, GT	Low

Abbreviations: CF: Clinical features, MH: Medical History, GT: Genetic testing, MS: Management and Support, E: Experiences, GC: Genetic counselling.

### Data synthesis

Narrative synthesis was utilised for this integrative review so that both quantitative and qualitative data were examined to answer the research question.^32,33^ A results-based convergent synthesis design was followed in which quantitative and qualitative data were synthesised simultaneously but independently and organised into key clinically relevant categories.^20^

## Results

### Study characteristics

There was a total of 109 reported patients with HDL2 across the 36 included papers. The total number of reported 109 patients with HDL2 in our review was slightly higher than the previously reported 91 cases by Krause et al. (2024),^1^ due to additional seven cases from Venezuela,^34^ four from South Africa,^35^three from Mali,^36^ two from Mexico,^37^ one from Brazil,^38^ and one from Italy (Supplementary Table 2).^39^ There were 26 quantitative studies, six qualitative studies, and four mixed-methods studies (Figure 1; Table 2). Most studies are effectively cross-sectional, even when designed as case-control studies or case series, due to the fact that patient assessments are typically conducted at a single point in time (Table 2). The studies originated from various countries across Europe, South America, Africa, North America, and Asia, with the majority from South Africa, mostly reporting on the same retrospective cohort, and Brazil (Table 2). Four articles had a high risk of bias, 14 had a moderate risk of bias and 18 had a low risk of bias (Table 2). Most of these studies focused on genetic and clinical characteristics, and medical histories of individuals with genetically confirmed HDL2 (Table 3). Most papers focused on diagnostic testing, while one South African study examined presymptomatic testing, identifying a single patient who received a positive presymptomatic result for HDL2 out of 57 individuals who requested testing for HD and HD phenocopies (Table 3).^40^ Seven studies explored the management of HDL2 symptoms, namely through medication,^12,14,36,39^^41–43^ while only five studies delved into the patient experiences of living with the condition.^35,36^^43–45^ No articles explored patient experiences of receiving genetic counselling for the condition, and no genetic counselling protocols for HDL2 have been reported in the literature. We do, however, note the use of genetic counselling sessions in some research protocols.^9,12,35,43^^46–48^ For instance, a South African paper stated that counselling was provided by trained genetic counsellors to all participants,^35^ while another noted that their patient from Botswana and his family had been counselled about the nature of the disease and prognosis; in both cases, sessions were included as part of the study protocol (Table 2).^12^ Sizer et al. (2012) noted that presymptomatic testing for HD and HDL2 in a South African setting was not always accompanied by comprehensive counselling, with only 38 of 57 individuals consulting a genetic counsellor, neurologist, psychologist, and psychiatrist before receiving their HD test results.^40^

**Table 3.** Demographic, genetic and clinical characteristics of HDL2, and main findings from all included papers, representing a total number of 109 cases with HDL2 (see supplementary Table 2).

Author	Demographic data	*JPH3* repeat length	Age of onset	Main method used	Comments
Anderson et al. (2017)^61^	Genetically diagnosed with HDL2, n = 12, genetically diagnosed with HD, n = 13, u naffected age matched controls n = 21. Population: African or mixed ancestry	Average: 46.3 Range: 43–53 (compared to average: 44.3, range: 40–49 for HD)	Not specified	Light microscopy assessment of erythrocyte morphology	No evidence of acanthocytosis in any of the HLD2 or HD patients. Duration of disease for HDL2: average 5.9 years, range 1–18 years (compared to average 8.2 years and range 4–18 years for HD)
Anderson et al. (2019)^9^	Genetically diagnosed with HDL2, n = 15, genetically diagnosed with HD, n = 13. Population: African or mixed ancestry	Median: 46 (equal to 46 for HD)	Median:41 years (compared to 40 for HD)	Unified Huntington's Disease Rating Scale (UHDRS)	Mean disease duration for HDL2: 4 years (compared to 7 years for HD) Movement-related symptoms were most prominent, followed by cognitive and psychiatric symptoms for HDL2. Initial symptoms were dementia, chorea, and abnormal eye movements, progressing to a stiffness and slow movement.
Anderson et al. (2019)^46^	Genetically diagnosed with HDL2, n = 9, genetically diagnosed with HD, n = 11, unaffected controls, matched for gender, age and race n = 9. Population: African or mixed ancestry	Average: 47 (compared to 44.9 for HD)	Average:42 years (compared to 39.4 for HD)	Brain MRI	Average disease duration: 5.6 years (compared to 6.6 years for HD) Similarity was noted between the MRIs of HD and HDL2 patients, with the exception that average thalamic volumes were smaller in the HDL2 group.
Baine, Krause and Greenberg (2016)^55^	Genetically diagnosed with HDL2, n = 52 (African ancestry n = 34, mixed ancestry n = 18)	Not specified	Not specified	Retrospective record review from 1995 to 2014 at UCT/NHLS and Wits/NHLS diagnostic labs	Minimum estimates of disease frequency in HD/HDL2 are: 5.1, 2.1 and 0.25 (per 100,000 individuals) for the European, mixed ancestry and African population groups respectively.
Bardien et al. (2007)^51^	Genetically diagnosed with HDL2, n = 3. Population: African or mixed ancestry	Range: 49–59	Range: 25–48 years	Genetic testing for pathogenic repeat expansions in *JPH3,* Brain MRI, EEG, Mini-mental state examination (MMSE), Neurological examination (details not specified)	Duration of disease not specified. All patients had dementia. III-17: Prominent chorea. Main symptoms were psychiatric (echolalia, disinhibited, aggressive) III-9: Predominantly Parkinsonian features. Dementia was the only psychiatric symptom. Normal cerebellum but head of the caudate was absent. IV-1: Tremor was initial symptom, followed by personality changes.
Bauer et al. (2002)^57^	Individuals referred for CAG/CTG repeat testing, n = 1600. Population: European ancestry	Range: 10–27	N/A	Genetic testing for pathogenic CAG/CTG repeat expansions in *JPH3*	None of the patients had an expanded allele in *JPH3* gene.
Bocoum et al. (2024)^36^	Genetically diagnosed with HDL2, n = 3 (2 males and 1 female) 28–56 years. Population: West African ancestry	40 (in all patients)	Range: 26–52 years	Brain MRI, Genetic testing for expansions in *JPH3,* Clinical examination performed by neurologist and psychiatrists, using clinical assessment scales, including UHDRS and MMSE.	Disease duration: 4 years (III-5 and IV-3), <1year (IV-4) III-5: Initial symptoms were involuntary movements. Main clinical features are generalised chorea, speech and movement impairment and irritability. Brain MRI showed bilaterial signal anomalies, cortical and basal ganglia atrophy. IV-3: Initial symptoms and main clinical features were speech impairment and mood disorder. IV- 4: Initial symptoms were unsteady movement. Main clinical features are chorea in the face area and hand tremors. 4 other family members reported with shakes, mood disorder and psychiatric symptoms but genetic test results not specified.
Castilhos et al. (2014)^59^	Genetically diagnosed with HDL2, n = 4. Population: African or mixed ancestry	Range: 47–56	Range: 29–44 years	Genetic testing for expansions in *JPH3,* Brain MRI, Clinical examination by neurologist or clinical geneticist	Duration of disease not specified. Family 38: Initial symptoms were mutism and cognitive decline, followed by psychiatric symptoms and movement abnormalities. Diffuse basal ganglia and brain atrophy. Father showed similar clinical symptoms. Family 57: AD family history. Initial symptoms were personality changes, memory loss and speech problems, followed by chorea. Mild atrophies. Family 375: Initial symptoms were cognitive decline and movement abnormalities, worsened over time. Father had similar clinical symptoms Family 397: Mother affected, same age of onset. Initial symptom was chorea, followed by mild cognitive decline
De Oliveira et al. (2020)^38^	Male, 67 years, Brazilian and African ancestry, genetically diagnosed with HDL2	44	63 years	Brain MRI, Neurological examination (details not specified), Genetic testing for expansions in *JPH3*	Disease duration: 4 years. Main symptom was movement abnormalities (initially chorea then parkinsonism). Epileptic seizures. No overt psychiatric symptoms. Prominent diffuse cortical atrophy.
Ferreira-Correia et al. (2020)^43^	Genetically diagnosed with HDL2, n = 7, unaffected controls matched by age, education level and language, n = 92. Population: African or mixed ancestry	Range: 43–53	Not specified	Montreal Cognitive Assessment (MoCA) total score, Rey Auditory Verbal Learning Test, Stroop Word-Colour Interference Test, Symbol Search Subtest, Letter-Number Sequencing subtest, Spatial Addition subtest, Visual Reproduction Subtests, Controlled Oral-Word Association Test, Design Fluency Test, Tower Test, Symbol Digit Modalities Test, Hooper Visual Organization Test, Purdue Grooved Pegboard Test	Disease duration range: 1–6 years. Movement speed, hand coordination, and visual-spatial skills susceptible to severe impairment. Executive function, attention and concentration susceptible to moderate impairment. Working memory susceptible to minimal impairment. 6/7 had anxiety/ sadness. 4/7 exhibited depression/ aggressive behaviour. 4/7 showed moderate global atrophy. 3/7 showed severe atrophy in the caudate.
Ferreira-Correia, Krause and Anderson (2020)^48^	Genetically diagnosed with HDL2, n = 15, genetically diagnosed with HD, n = 13. Population: African ancestry or mixed ancestry	Mean: 47.2	Mean: 41.5 years	Clinical assessment by neurologist, using UHDRS, Interviews with patients and unaffected informants	Mean duration of disease 5.1 years. HDL2 patients reported non-motor symptoms as the first sign of the disease more frequently, compared to HD patients who report motor symptoms. Disruptive behaviours, depression, anxiety, and irritability were most frequent symptoms for HDL2. Hallucinations were rare, and no suicidal ideation.
Ferreira-Correia et al. (2022)^47^	Genetically diagnosed with HDL2, n = 3, g enetically diagnosed with HD matched by age, years of education, schooling and language, n = 7, unaffected controls matched by age, years of education, schooling and language, n = 92. Population: African or mixed ancestry	Range: 44–53	Range: 31–48 years	Same methods previously outlined in Ferreira-Correia et al. (2020)^43^	Disease duration range: 1–6 years. No significant differences were observed between the two disorders except: HDL2 cases show significant problems with working memory. HD cases do not struggle with working memory but struggle with delayed retrieval of auditory/verbal information.
Fischer, Licht and Mendez (2012)^14^	Male, 63 years, African ancestry, g enetically diagnosed with HDL2	43	55 years	Brain MRI, Fluoro-deoxyglucose (FDG) positron emission tomography (PET) brain scan, Genetic testing for expansions in *JPH3,* MMSE, MoCA, Digit-Span Forward, Digit-Span Reverse, Months-in-Reverse, “A” Vigilance Test, Serial “3's” Test, Verbal Fluency, Mini-Boston Naming Test, Auditory Verbal Learning Test, Delayed recall, Recognition, Visuospatial, Frontal Assessment Battery, Idioms and Proverbs	Duration of disease: 8 years. Positive family history for Parkinson-like problems. First symptom was walking and balance issues, followed by neuropsychiatric symptoms. Personality change (irritability and aggression) developed as condition worsened. Caudate nuclei on both sides of the brain were barely visible. Basal ganglia appeared very thin. Decreased metabolic activity in both the caudate nuclei and putamen.
Greenstein et al. (2007)^45^	Genetically confirmed HDL2, n = 3. Population: Caribbean and African ancestry	Range: 43–59	Range: 18- 54 years	Chart review, Neuropathology using immunoperoxidase stains, Genetic testing for expansions in *JPH3,* Brain MRI, CT scan	Disease duration range: 20–22. Brain weight reduced by 15%. Caudate nucleus had undergone the greatest neurodegeneration. Case 1: Initial symptoms were personality changes and paranoid behaviour, followed by speech difficulties. Progressive cognitive decline and Parkinsonian symptoms developed after. Positive family history for similar clinical features. Case 2: Initial symptoms were depression, social withdrawal, and rapid weight loss. Walking and speech difficulties followed. No paranoid ideations, hallucinations, or chorea. Positive family history, demonstrating anticipation.
Ibanez et al. (2024)^91^	Genetically diagnosed with HDL2, n = 1	Not specified	Not specified	Analysis of DNA repeat expansion loci including *JPH3* in 100,000 Genomes Project (100 K GP) and Trans-Omics for Precision Medicine (TOPMed)	Among a cross-sectional cohort of 82,176 people, one individual was identified with an allele in the pathogenic full mutation range
Krause et al. (2015)^10^	Genetically diagnosed with HDL2, n = 41 (32 African ancestry, 9 mixed ancestry), unaffected controls (African ancestry n = 176, White ancestry n = 134)	Range for HDL2: 40–58, range for controls: 6–26	Not specified	Genetic testing for expansions in *JPH3*, Retrospective file analysis, *JPH3* haplotype analysis	Fewer HDL2 patients have a positive family. Chorea, dementia, and emotional disturbance in 45% of HDL2 patients. Parkinsonian features more prominent in HDL2 than HD patients. Caudate atrophy and generalised cerebral atrophy.
Magazi et al. (2008)^52^	Genetically diagnosed with HDL2, n = 6	Not specified	Range: 40–64 years	Genetic testing for expansions in *JPH3,* clinical assessment by neurologist, Mayo Mini Mental test, brain imaging	3/6 had a positive family history. Chorea was present in all patients. Later age of onset for chorea in HDL2
Margolis et al. (2004)^49^	Genetically diagnosed with HDL2, n = 26. Population: African or mixed ancestry	Range: 44–57	Not specified	Genetic testing for expansions in *JPH3*	Length instability in maternal transmission detected at 33 repeats. HDL2 repeat length: 35 (two triplet increase in proband's son) Younger age of onset correlated with longer repeat length.
Mariani et al. (2016)^92^	Genetically diagnosed with HDL2, n = 3. Population: Caribbean, African or mixed ancestry.	Not specified	Not specified	Brain MRI, Genetic testing for expansions in *JPH3*, UHDRS, clinical examinations (details not specified)	All had a positive family history, behavioural or mood disorder and cognitive impairment. Most had dystonia and parkinsonism (2/3). All had brain atrophy (2 global, 1 caudate)
Mulroy et al. (2020)^60^	Female, 51 years, African ancestry, genetically diagnosed with HDL2	52	45 years	DAT-SPECT imaging, genetic testing for expansions in *JPH3,* clinical examinations (details not specified)	Disease duration: 6 years. Initial symptoms were movement abnormalities, balance, and memory issues. Followed by mild generalised chorea and changes in posture. Positive family history - mother and brothers showed similar symptoms. Reduced level of dopamine activity in the striata
Narotam-Jeena et al. (2024)^35^	Genetically diagnosed with HDL2, n = 8 (4 deceased). Population: mixed ancestry	Median: 52 (range: 45–63)	Median: 38 years (range: 18–48)	Brain MRI, UHDRS, MoCA, MMSE, DNA analysis for expansions in *JPH3*	Disease duration not specified. Initial symptoms were cognitive (more severe) and motor impairments. All patients presented neuropsychiatric symptoms (behavioral change, memory impairment, depression, anxiety/agitation, irritability, hallucinations). All patients showed caudate atrophy and generalised cerebral atrophy. 6/8 had generalised dystonia, 6/8 had chorea (mild and restricted to toes, arms, trunk and face areas).
Nguyen et al. (2022)^56^	Physicians participating in survey, n = 20. Population from Europe, USA, Australia and China.	N/A	N/A	Literature review of 51 articles from 1993 to 2020 from Pubmed about chorea or HD-like disorders, Survey	75% of clinicians voted African ancestry as an indication of HDL2. Clinicians also voted caudate head and basal ganglia atrophy as a HDL2 red flag (% not specified). Literature review identified acanthocytosis and myoclonus as an indication of HDL2.
Ocampo, Daimari and Oyekunle (2018)^12^	Male, 47 years, Botswana	49	∼40 years	Clinical examination (details not specified) Brain MRI, Genetic testing for expansions in *JPH3*	Disease duration: 4 years. Chorea, movement abnormalities, slurred speech, mood instability, behavioural changes, cognitive impairment, and weight loss. Normal eye movements. No rigidity. Father and paternal aunt had similar movement disorders. Cortical and bilateral atrophy of the caudate nucleus.
Paradisi, Ikonomu and Arias (2013)^50^	Genetically diagnosed with HDL2, n = 4. Population: Venezuelan (mixed) ancestry	Range: 39–47	Range: 38–55 years	Genetic testing for expansions in *JPH3* using HDL2f and HDL2r primers, with genotyping of polymorphic markers, case report (no details provided).	All had similar clinical manifestations at onset: personality changes (aggressiveness), insomnia and depression, shortly followed by involuntary movements. 3 of 4 carried the African marker Duffy null.
Paradisi, Ikonomu and Arias (2024)^34^	Genetically diagnosed with HDL2, n = 11	Range: 39–47	Range: 38–66 years	Genetic testing for expansions in *JPH3,* Locus-specific DNA amplification and haplotype markers, Record review	Disease duration not specified. Minimal prevalence figures: Yaguaraparo: 60 per 100,000 families, Puerto Cumarebo: 31 per 100,000 families, Boca del Tocuyo: 36 per 100,000 families. 7/11 showed psychiatric symptoms, chorea, dementia and involuntary movements at disease onset. 7/11 carried African marker duffy
Ramirez-Garcia et al. (2022)^37^	Genetically diagnosed with HDL2, n = 2	44 and 45 (DNA repeat length was also within the intermediate range for HD: 27 and 28)	36 years	Examination of medical records for clinical information, genetic testing for expansions in *JPH3,* clinical examinations	Disease duration not specified. Clinical features: irritability, aggressiveness, anxiety, depression, cognitive impairment, movement abnormalities. and striatal atrophy
Rodrigues et al. (2008)^42^	Genetically diagnosed with HDL2, n = 4. Population: confirmed and probable African ancestry	Range: 46–59	Range: 22–60 years	Genetic testing for expansions in *JPH3*, Neuroimaging and clinical examination (no details included)	All had dementia and movement disorders (chorea and parkinsonism most prominent). All had psychiatric disturbance (depression and aggressive behaviour most prominent). All had caudate atrophy. Family history of chorea, dementia, or psychiatric disturbance.
Rodrigues et al. (2011)^93^	Genetically diagnosed with HDL2, n = 3. Population: Brazilian	Not specified	Not specified	Medical records review from 1998 to 2006, Genetic testing for expansions in *JPH3* using PCR primers	Among 29 Brazilian patients with a HD-like phenotype, HDL2 occurred in 3.
Ruscitti et al. (2022)^39^	Female, 51 years, Brazilian ancestry	46	Not specified	Neurological examination, including Luria test, MMSE, free and cued selective reminding test, clock drawing test, constructive apraxia test, symbol digit and stroop test, CT scan, Blood smear, genetic testing for expansions in *JPH3,* fluorodeoxyglucose PET scan, fibroendoscopic examination, fiberoptic endoscopic evaluation (FEES)	Negative family history. Initial symptoms were involuntary movements, CT scan of brain was normal. Posture and walking problems followed, then agitation and confusion. Speech and swallowing problems were gradually compromised. Reduction in brain activity in basal ganglia.
Santos et al. (2008)^58^	Male, 48 years, Brazilian and African ancestry, non-consanguineous parents	47	44 years	Clinical evaluation, including UHDRS and brain MRI, haplotype studies using insertion/deletion polymorphisms method, genetic testing for expansions in *JPH3*	Father showed similar clinical features at 50. Proband's five siblings and three offspring are asymptomatic. Initial symptom was delayed reaction time, followed by slurring speech, movement abnormalities and progressive decline in cognitive function. Overall brain atrophy – diffuse in basal ganglia and severe in caudate head
Sizer et al. (2012)^40^	Individuals who had diagnostic testing for HDL2, n = 19, individuals who had presymptomatic testing for HDL2, n = 1	Not specified	Not specified	Retrospective record review from 1998 to 2006	Positive HDL2 results: 84.2% had African ancestry and 15.8% had mixed ancestry. No White or Indian patients.
Stevanin et al. (2003)^53^	Genetically diagnosed with HDL2, n = 2. Population: African (Caribbean and Moroccan) ancestry	Range: 43–50	Range: 42–51 years	Genetic testing for expansions in *JPH3*, clinical assessment by movement disorder specialists	Negative family history. Initial symptoms were behavioural changes, memory loss and chorea. Other symptoms were dementia, decline in higher cognitive processes. No parkinsonism or eye abnormalities. Subcortical, cortical, and caudate nucleus atrophy.
Vasconcellos et al. (2017)^41^	Male, 36 years African ancestry	52	Not explicitly stated but inferred to be 23 years	Clinical examination (no details provided), MMSE, MoCA, UHDRS, peripheral blood smear, brain MRI	Disease duration: 13 years. Movement abnormalities were prominent (initially bilateral and asymmetrical) Other symptoms were memory problems, weight loss, impaired cognition. No vision problems. No acanthocytes. Caudate and lentiform nuclei atrophy. Patient's father and cousin had dementia and chorea.
Walker et al. (2003)^44^	Genetically diagnosed with HDL2, n = 5	Range: 44–58	Range: 26–45 years	Genetic testing for expansions in *JPH3*, Brain MRI, Clinical examination, including MMSE.	Overall brain atrophy – severe in caudate and putamen. Most prominent symptoms were dementia, chorea, and depression. Parkinsonism, dystonia, and acanthocytosis were less frequently observed. Patient 2: Disease duration: 9 years. Father and grandfather showed similar symptoms.
Walker et al. (2003)^11^	Genetically diagnosed with HDL2, n = 9. Population: African/ Mixed, Mexican ancestry	Range: 44–58	Range: 12–55 years	Genetic testing for expansions in *JPH3*, Acanthocytosis and red cell membrane electrophoresis	Chorea was most prominent symptom (8/9). Other movement abnormalities present but less prominent. Dementia in 6 patients, 3 had depression, 4 had acanthocytosis.
Wild et al. (2008)^54^	Female, age unknown, genetically confirmed HDL2	Not specified	41 years	Genetic testing for expansions in *JPH3*, clinical case descriptions	No family history. No movement abnormalities, vision or speech problems, Psychiatric symptoms (irritability, aggression, depression) and cognitive decline present since onset. Chorea and dystonia developed subsequently. Global cortical atrophy.

### DNA repeat length and impact on phenotype

DNA repeat length was reported in 28 articles, with most cases indicating pathogenic repeat lengths starting at 40 repeats and extending into the 50 s (Table 3). Narotam-Jeena et al. (2024) examined eight HDL2 cases in Cape Town between January 2006 and September 2021 and reported the highest number of DNA repeats as 63.^35^ Three studies addressed the uncertain significance repeat range, which is thought to be between 29–39 repeats, where symptoms may or may not develop. Interestingly, one article from the USA investigated repeat length stability for a mother who had a repeat expansion of 33 DNA repeats.^49^ In this case, maternal transmission led to a two-triplet increase from mother to son.^49^ Initially diagnosed with an atypical cerebellar disorder, the mother showed minimal progression in cerebellar symptoms five years after the initial evaluation at age 53.^49^ Her son, who exhibited concentration issues, balance problems, and eye movement abnormalities by age 30, had a 35-repeat expansion compared to his mother's 33 repeats.^49^ However, the authors concluded that the son's symptoms were unlikely due to the number of repeats, given that his mother's symptoms did not follow the slowly progressive pattern typically seen in HDL2 patients and an alternative explanation of autoimmune Cogan's syndrome perhaps accounting for the son's clinical features.^49^ Another Venezuelan patient was identified as having an expanded allele at the upper end of the uncertain significance repeat range, an uninterrupted CAG/CTG stretch of 39 repeats, which led to late clinical manifestations at 55 years of age.^34,50^ In contrast, Krause et al. (2015) examined the normal allele distribution at the *JPH3* locus in unaffected controls in South Africa.^10^ They found that normal repeat lengths ranged from six to 26 and that this number was similar in individuals with both European and African ancestry with no apparent skewing.^10^

### Age of onset and disease duration

Age of onset was reported in 25 articles, with most patients developing initial symptoms in their 40 s or early 50 s (Table 3). Patients with onset in the mid-20 s were reported in five studies.^11,35,36,44,51^ Greenstein et al. (2007) conducted a mixed-methods study in the USA, using a clinical case description regarding a 34-year-old patient with HDL2 whose son, with 59 repeats, developed symptoms at age 18.^45^ However, details about the son's genetic testing and clinical diagnosis were not specified.^45^ Narotam-Jeena et al. (2024) was the only study to report diagnostically confirmed juvenile-onset HDL2, describing two patients (age of onset at 18 and 19) with large repeat expansions (63 and 59 repeats, respectively).^35^ These studies suggest that a higher number of repeats is generally correlated with an earlier age of onset. Narotam-Jeena et al. (2024) hypothesised that large expansions may result in the patient developing dementia, dystonia, and rigidity.^35^ However, no other studies established a clear link between repeat length and clinical presentation. Interestingly, one study found that symptomatic patients subsequently diagnosed with HDL2 sought a diagnosis on average three years sooner than patients with HD.^43^

Disease duration was included in 12 studies, with the majority indicating a four to five-year course.^9,12,36,38,43^^46–48^ This may not accurately represent the time from symptom onset to death, as some patients were still living when data collection ended. Some articles investigated patients who had a longer duration of symptoms, including one article reporting a patient who has been living with HDL2 for 22 years since disease onset.^45^

### Family history

A positive family history was variable among the patients who participated in these studies. Four articles observed that patients had no previous family history of movement disorders or psychiatric disturbances.^39^^52–54^ A case report by Ruscitti et al. (2022) stated a negative familial history of neurodegenerative diseases in the 51-year-old Brazilian proband, who was the first case of HDL2 reported in Italy.^39^ Stevanin et al. (2003) is a cross-sectional study from France examining 252 people with a Huntington's disease-like (HDL) phenotype, two of whom were identified to have HDL2 with DNA variants in *JPH3* within the pathogenic range (43 and 50 uninterrupted CTG repeats).^53^ One of the two patients mother was a non-carrier and their father died before the age of 60 from cardiac arrest; the other individual's parents had not been tested – their father died aged 54 and their mother was alive (age and history not reported).^53^ Wild et al. (2008) reported a case series including one HDL2 patient from the largest reported cohort of HD phenocopy patients in the UK, and the article does not elaborate on the absence of this patient's positive family history.^54^ Magazi et al. (2008) presented a South African case series identifying six patients with HDL2.^52^ While three of these patients had a positive family history, the authors noted an inability to establish a positive family history for two patients and reported an uncertain family history for the final patient.^52^ Articles reporting a positive family history based this assessment on relatives exhibiting a similar clinical picture, such as dementia, chorea, and other movement disorders, without molecular confirmation of HDL2. Interestingly, Krause et al. (2015) found that a positive family history was reported in 84% of HD families compared with only 50% of HDL2 families, based on a study of 39 HD patients and 22 HDL2 patients.^10^

### African ancestry

Several studies have investigated the prevalence, genetic ancestry, and geographic distribution of HDL2 in affected individuals.^34,50,55^ A Venezuelan study by Paradisi, Ikonomu and Arias (2024) analysed data from 260 independent index cases referred to the Human Genetics Laboratory between 1985 and 2022.^34^ They identified HDL2 in 11 families and estimated the minimum population prevalence within geographic clusters across Venezuela.^34^ Their findings indicated a prevalence ranging from 31 to 60 per 100,000 families, with notable clustering in Yaguaraparo, Puerto Cumarebo, and Boca del Tocuyo.^34^ African ancestry seems to have been relevant for all patients with HDL2 in the studies to date. In a survey aiming to identify indications to consider before ordering HDL2 testing in France, 75% of clinicians specified that African ancestry is a primary clinical indicator for HDL2.^56^ In four unrelated Venezuelan families with HDL2 (with no known African ancestry), three carried the African marker Duffy null *ACKR1* blood group antigen genotype commonly found in individuals of African descent.^50^ Conversely, the incidence of HDL2 among people of non-African ancestry seems to be extremely low. In an American study analysing CAG repeats in a cohort of 1600 patients of presumably self-reported European ancestry, 147 patients had a reported family history of choreatic symptoms and negative HD results, and HDL2 genetic testing was also negative, with CAG repeat sizes ranging from 10 to 27 units.^57^

### Neurological characteristics

Twenty-two studies aimed to characterise the neurological profile of HDL2 (Table 3). Brain atrophy was consistently reported across all 22 studies, with some noting generalised cerebral atrophy in HDL2 patients,^10,35,42^ while others identify the most severe atrophy in the caudate nucleus.^11,43,45,58^ One case report from the US noted that the caudate nuclei were barely visible on both sides of the brain.^14^ Additionally, the basal ganglia were also observed to have neurodegeneration^14,36,39^; however, this was generally labelled as diffuse atrophy.^58,59^ Interestingly, the survey conducted by Nguyen et al. (2022) also found that clinicians recognised caudate head and basal ganglia atrophy as indicative of HDL2, although the exact percentage was not reported.^56^ Narotam-Jeena et al. (2024) reported leukoaraiosis in four of their seven patients who had brain magnetic resonance imaging performed.^35^ Mulroy et al. (2020) noted a reduced level of dopamine activity in the striata, a component of the basal ganglia involved in movement control.^60^ Another noteworthy finding was that the average thalamic volume was 21% smaller in HDL2 patients compared to HD patients, even when controlling for various factors such as age at the time of MRI, disease duration, abnormal repeat length, and age of disease onset.^46^

### Clinical characteristics

Thirty-three articles provided insight into the clinical presentation of HDL2, describing a range of initial symptoms, including dementia,^9,34^ personality changes,^12,45,50,59^ and chorea,^9,34,36,38,42,51,53,59^ as the most prominent symptoms. While symptoms varied widely, dementia emerged as a consistent and prominent feature across studies.^9^Cognitive decline has been observed to start early in HDL2 disease progression, evolving into dementia with variable features.^43^ Examining seven patients in South Africa, this study noted varied patterns of cognitive decline, with one patient struggling specifically with the immediate recall of visual data, another displaying a more generalised visual memory deficit, and two patients showing no significant impairments in visual tasks.^43^ Interestingly, this study also highlighted prominent psychiatric symptoms, such as disruptive behaviours, depression, anxiety, and irritability, with rare occurrences of hallucinations.^43^ Chorea, movement abnormalities, speech issues and cognitive decline were also noted, although the frequency and severity of these symptoms varied between studies.

Variability in symptoms is evident, with some studies indicating psychiatric symptoms as predominant, while others emphasise movement abnormalities. Narotam-Jeena et al. (2024) reported predominantly cognitive and behavioral presentation at onset in patients with large repeat sizes (45–63 repeats).^35^ They noted that while patients may have exhibited concurrent motor symptoms, cognitive and behavioral features were more pronounced.^35^ This was evident in all but one patient scoring below 15 on cognitive screening tests such as the Mini-Mental State Examination and Montreal Cognitive Assessment.^35^ Additionally, early psychiatric symptoms were common among these eight patients, ranging from depression and psychosis to behavioral changes, including agitation and irritability.^35^

De Oliveira et al. (2020) presented a qualitative case report of a 67-year-old male Brazilian HDL2 patient, identifying movement disorder, particularly chorea followed by parkinsonism, as the primary symptoms, with no overt psychiatric manifestations.^38^ Similarly, qualitative case report from Mulroy et al. (2020) identified a 51-year-old HDL2 patient of self-reported Afro-Caribbean ancestry in the UK, whose initial symptoms included movement abnormalities, balance issues, and memory difficulties, later progressing to mild generalised chorea and postural changes.^60^ In both cases, no overt psychiatric symptoms were observed.^38,60^

Castilhos et al. (2014) conducted a retrospective case series analysing data from 104 families ascertained between 2001 and 2012 at neurogenetics centers across seven urban locations in Brazil, including four patients with HDL2.^59^ The study found that while movement-related symptoms were most prominent, cognitive symptoms were also observed in all four of these four patients.^59^ These findings are supported in a 2024 study, which identified intra-familial variability in symptoms among three affected individuals, all with 40 repeats and an age of onset ranging from 26 to 52 years.^36^ Notably, the father (proband) primarily exhibited motor symptoms, including uncontrollable movements in his face, limbs, and trunk, leading to difficulty walking.^36^ In contrast, psychiatric symptoms were predominant in his daughter, who had a mood disorder and a history of multiple suicide attempts.^36^ This variability extended to other family members, with the proband's paternal grandmother presenting with tremors while his older sister presented with a mood disorder, though it remains unclear whether the latter has been diagnosed with HDL2.^36^

Psychiatric symptoms preceded movement symptoms in multiple patients.^34,45,48,50,53,54,59^ Paradisi, Ikonomu and Arias (2024) identified psychiatric symptoms as one of the most common initial manifestations, observed in seven of their eleven HDL2 patients.^34^ Presenting psychiatric symptoms preceding motor symptoms included personality changes, paranoid behaviour, depression, and social withdrawal,^45^ depression and insomnia,^34^ irritability, aggression, depression,^54^ apathy, irritability, and difficulty with social integration,^36^ and anxiety and depression.^35^

Acanthocytosis was identified as a clinical feature in some HDL2 patients. A literature review by Nguyen et al. (2022) conducted as part of a broader study for clinical indications for identifying HD phenocopies highlighted acanthocytosis and myoclonus as features for physicians to consider regarding a potential diagnosis of HDL2.^56^ While some studies found no defined acanthocytosis or morphological abnormalities,^41,61^ other articles reported the presence of acanthocytosis in some patients.^11,44^

### Experiences and support

No papers specifically reported on patient perceptions or experiences of genetic counselling.

Experiences of employment loss and high care needs were reported in the few studies which provided information on patient perspectives, but data on this is limited. Five studies, consisting of four case series and one cross-sectional study, mentioned the experiences of individuals living with HDL2.^35,36,44,45,48^ Out of these five studies, only Ferreira-Correia, Krause and Anderson (2020) conducted clinical interviews for their 15 HDL2 patients in South Africa as well as an unaffected family member or caregiver to obtain biographical and medical history.^48^ This article, which also incorporated a Unified Huntington's Disease Rating Scale (UHDRS) functional assessment for their patients, noted that some patients needed full-time assistance with the activities of daily living, but none were able to access appropriate services.^48^ The remaining four articles relied on different sources for this information, such as patient medical records;^45^ UHDRS results;^35^ a clinical examination conducted by a multidisciplinary team, including neurologists and psychiatrists;^36^ and a comprehensive assessment that included videotaped documentation of characteristic features.^44^

Greenstein et al. (2017) described two patients, one of whom was unable to continue working as a cafe owner when his symptoms of paranoid behaviour and personality changes started at age 54.^45^ His escalating psychiatric symptoms and combative behaviour eventually necessitated full-time care in a nursing home.^45^ The second patient, a stock room worker, was dismissed from work due to movement and speech abnormalities.^45^ Over time, his condition deteriorated, resulting in mutism five years post-presentation and becoming bedridden and cognitively impaired, with reliance on a feeding tube 14 years after the initial onset.^45^ Walker et al. (2003) was a case series that clinically examined five patients and detailed a 54-year-old patient who experienced personality changes, depression, and financial management difficulties, resulting in the loss of his job in a highly skilled profession.^44^ Narotam-Jeena et al. (2024) observed that among their eight participants, difficulty working and managing personal affairs was a common challenge.^35^ While some were still able to attend to personal hygiene and eat without assistance, most received care at home, though specific details regarding the type of care and support were not provided.^35^ One participant was able to perform household chores and supervise children.^35^ Bocoum et al. (2024) highlighted how psychiatric management for severe depression was required for one of their HDL2 patients, following multiple suicide attempts.^36^ The remaining articles, including Bocoum et al. (2024), underscored the extensive medical management needs of HDL2 patients, including psychiatric hospital admissions and intermittent use of antipsychotic medications and other pharmacologic treatments which may or may not prove effective.^12,14,36,39,41,42,48^

## Discussion

This systematic review has synthesised existing evidence related to HDL2 to inform the process of genetic testing and counselling, and diagnosis of HDL2. This is a rare condition with reports of only 109 patients with HDL2 found in the literature.^1^ Here, we have synthesised key information about DNA repeat lengths, age of onset, family history considerations, disease duration, as well as neurological and clinical characteristics are summarised.

Consistent with other studies, our review has identified significant variability in HDL2 symptoms among patients.^1, 62,63^ The average age of onset for HDL2, 41–42 years, is similar to that observed in Huntington's disease (HD).^13,64^ However, data to clarify the variability in age of onset and symptoms and correlation with the *JPH3* repeat numbers are lacking.

Several articles from our review suggest that family history was variably reported among patients with HDL2.^10,39^^52–54^ Notably, family history appears to be documented less frequently in HDL2 compared to HD.^10^ While this may reflect an absence of familial history or possible de novo cases, Krause et al. (2015) highlight additional contributing factors, including poor history taking or ascertainment, reduced lifespan, later age of onset, and variable phenotype.^10^ Without data to clarify this discrepancy, these considerations are important in interpreting the apparent differences in reported family history between HDL2 and HD.

Current literature continues to emphasise the prominence of chorea and movement disorders, with variable dementia, cognitive and psychiatric symptoms.^1,15,65^ Dementia and cognitive decline have also been reported in previous systematic reviews investigating HDL2.^1,15,65^ Neurocognitive assessments often include standardised scales such as MOCA and MMSE.^9,14,35,36,39,41,43,44,47,48,51,52^ However, cognitive features are not consistently well documented in many studies, as shown in Table 1 from Ferreira-Correia et al. (2020).^43^ In the studies mentioned, cognitive decline is often noted without specifying the assessment method used to reach this conclusion.^43^ This paper highlights that the reliance on descriptive, unspecified, or variable assessments has further limited the strength of the evidence in this area.^43^ The authors also note that an accurate neuropsychological characterisation of HDL2 patients can be influenced by various factors, including level of education and multilingualism, which may impact cognitive performance.^43^ Overcoming practical barriers to performing the assessments including those relating to social inequality and linguistic diversity are important considerations.^43^ Addressing these factors may improve the reliability of cognitive assessments in HDL2, allowing for a clearer understanding of the condition's neuropsychological profile and its progression over time.

Some studies suggest that psychiatric manifestations often precede other symptoms in HDL2 which raises the possibility that the onset of psychiatric symptoms without accompanying movement disorders may cause misdiagnosis and delay the diagnosis and management of HDL2.^48^ Although descriptions of HDL2's neuropsychiatric symptoms exist, they are often inadequately characterised in the literature. Most reports of neuropsychiatric symptoms are brief, incomplete, and often embedded within clinical case descriptions.^12,39,42,44^ Information about potential limitations or biases were often omitted.

Several articles provided details on how clinical examinations were conducted by a multidisciplinary team, including neurologists and psychiatrists.^36,44,59^ However, reporting perceptual symptoms of neurological patients can be challenging for neurologists and psychiatrists.^66^ The complexity of these symptoms, combined with the absence of readily available objective measures for diagnosis, adds to the difficulty.^66^ Trapp et al. (2022) propose a biopsychosocial framework for obtaining a neuropsychiatric history, incorporating screening questions that assess symptoms along with their severity, acuity, and duration.^66^ This approach also considers factors such as trauma history, cognitive baseline, and substance use.^66^ This paper explored the use of psychiatric assessment tools as a standardised method of reporting symptoms that patients may struggle to articulate or that present differently across individuals.^66^ Another challenge in assessing neuropsychiatric symptoms is the potential for informants, whether patient or collateral informant, to overestimate or underestimate self-reported symptoms.^67^ Emotional distress and caregiver burden may influence their perception, impacting the accuracy and reliability of the report.^67^

Attention to psychiatric symptoms, alongside motor and cognitive signs, is essential for more accurate and timely diagnosis and treatment. Hence, this review underscores the importance of future research to better characterise the psychiatric manifestations of HDL2, and for multidisciplinary team members to consider HDL2 in their differential diagnosis of patients presenting with neuropsychiatric symptoms, even when motor abnormalities are absent.

This systematic review identified studies on HDL2 from a variety of countries, including South Africa, Brazil, Venezuela and across Europe. The findings of this review suggest that HDL2 seems to exclusively occur in individuals with definite or likely African ancestry. Krause et al. (2024) recently focused on this pattern in a systematic review but noted that no cases of HDL2 have been described in West Africa, possibly reflecting limited access to genetic testing and healthcare services.^1^ Our review identified one study reporting the first documented case of HDL2 in West Africa, suggesting that the condition may be more widespread across the continent, with increased access to genetic testing potentially revealing additional cases.^36^ An awareness of the prevalence in different populations will also be beneficial in advocating for and facilitating access to HDL2 genetic testing, thereby avoiding delays or inaccurate diagnoses.^34^ It is therefore important to ask about ancestry when gathering family history.

The *JPH3* gene could ideally be included in neurological, Parkinsonian, and chorea gene panels due to phenotypic overlap, especially in regions with many patients of African ancestry. However, incorporating *JPH3* testing into gene panels is challenging because conventional massively parallel sequencing (Next Generation Sequencing (NGS)) panels and exome tests often are unable to accurately detect and quantify repeat expansions, currently necessitating the use of gene-specific PCR testing.^68,69^ New bioinformatic analyses methods for short tandem repeats on NGS data, and alternative test methods such as long-read sequencing are starting to become available for diagnosis of DNA repeat disorders.^70,71^ Although PCR is generally considered simple and cost-effective, individual laboratories often lack the financial incentive to develop and validate infrequently used tests for rare conditions.^72,73^ Therefore, while the clinical integration of HDL2 testing is promising, access to an accredited test for a rare condition like HDL2 is difficult.^73^ Improved access will require collaborative efforts to develop standardised protocols and secure funding for equitable health care for families with HDL2.

There is minimal research on the implications of repeat lengths within the uncertain significance repeat range.^49,50^ In the case of HD, a reduced penetrance allele is thought to increase an individual's susceptibility to environmental stresses, such as oxidative stress or inflammation, by disrupting normal cell functions.^74^ A similar mechanism may also apply to HDL2, however, more research is required to confirm this hypothesis. A recent French study of individuals carrying 36–38 CAG repeats within the *HTT* gene highlights the potential for underdiagnosis due to the absence of chorea when repeat lengths fall below the currently defined full penetrance threshold.^75^ The authors demonstrated that patients within this range can be as severely affected as those with moderately larger expansions (40–42 CAG repeats), presenting with symptoms such as impaired episodic memory despite lacking typical motor features.^75^ Additionally, findings from a US study suggest that even intermediate alleles (27–35 CAG repeats), though not directly pathogenic, may nonetheless be associated with behavioural phenotypes.^76^ Thus, the apparent under-ascertainment of patients within the uncertain significance repeat range may reflect their non-specific phenotype rather than low penetrance alone.^75,76^ In HDL2, the clinical impact of alleles with fewer than 40 repeats likewise remains uncertain, underscoring the importance of further study to clarify their contribution to disease expression, refine diagnostic criteria, and guide accurate genetic counselling.

Information about repeat length instability and anticipation (the potential increase in DNA repeat length from one generation to the next) is also lacking.^49,50^ Drawing from HD literature, we know that understanding the effect of DNA repeat length ranges, especially regarding reduced penetrance alleles, is critical information for patients to understand their pre-test risk and manage the associated uncertainty.^3,77^ It assists interpretation of test results, provides avenues for targeted therapies, and supports psychotherapeutic counselling for adjustment to results and meaning-making processes in the context of the age-related risks, variability, uncertainty and hope, and questions about family planning.^3,77,78^ Accurate information and genetic counselling strategies can thereby empower individuals to make informed decisions which are congruent with the patient's values, priorities and present and future concerns.^79^

There are significant gaps in the literature regarding genetic counselling and the testing process for HDL2. No articles provide insights on genetic counselling protocols or explore patient experiences with HDL2 genetic counselling. International guidelines for HD presymptomatic testing have been developed.^80^ Given the similarities between HD and HDL2, these results suggest that using HD protocols is likely to be appropriate in the HDL2 setting.^80^ Close attention is required for accurate discussion regarding the HDL2 genetic implications and types of results, motivations behind testing, psychological and familial impact, prenatal and IVF test options (including availability of HDL2-specific testing), coping strategies and resources for families with HDL2, to ensure as much patient support as possible can be provided.^78,80^

This systematic review identified gaps in knowledge concerning patient and family experiences of HDL2. While articles were able to highlight challenges such as maintaining employment,^35,44,45,48^ and the level of care patients needed after symptom onset,^35,36,45,48^ none have set out to specifically explore and document the views, concerns, and challenges for individuals and families in a rigorous way. Drawing on the literature for HD provides parallels, which we anticipate may be similar for HDL2. For example, semi-structured qualitative interviews were conducted with 36 individuals from Norway, all above the age of 12, who had experience growing up with a parent with HD.^81^ The findings revealed that individuals perceive HD as a thief of relationships, as changes in personality, mood and behaviour altered the true nature of affected parents; of self, as HD triggers distressing thoughts about genetic status and life choices; and of transparency, as HD creates family rifts and secrecy about disclosure.^81^ Insight into patient experiences and familial dynamics is invaluable for addressing each aspect in counselling sessions.^82–84^ For example, a study on the psychosocial impact of presymptomatic HD testing on support persons found the experience most intense for at-risk offspring of probands.^82^ This highlights the need for more focused counselling for support persons in HDL2. The same study revealed an additional complexity for relatives who are at-risk themselves when managing caregiving after a positive result is declared.^82^ It may be useful for genetic counsellors to offer to talk one on one with the support person to address their concerns.^82^ However, more data is needed regarding the challenges, concerns, and needs of patients and families with HDL2 across various cultural contexts.

## Limitations

This review has several limitations related to both the individual articles and the review methodology. It included seven case reports and 13 case series, which are not traditionally part of systematic reviews due to their small sample sizes, selection bias, and lack of control groups.^85^ These factors limit the generalisability of findings, may not represent the typical patient experience, and prevent drawing definitive conclusions.^86^ However, given the limited data available on HDL2, these studies provide valuable information about the condition. Critical appraisals reveal that four articles had a high risk of bias, 14 had a moderate risk of bias and 18 had a low risk of bias. Due to the limited data and the rarity of HDL2, articles with a high risk of bias were not excluded and their findings were generally supported by other sources. In addition to methodological limitations, there were also inconsistencies in the data reported across the included articles. For example, distinguishing between young-onset dementia (<65 years) and late-onset dementia is crucial when assessing the likelihood of HDL2 or other genetic conditions.^87^ However, some articles either omitted the age of dementia onset or included late-onset cases as contributors to a positive family history. This systematic review only considered articles written in English, due to limited data and resource constraints. Despite these limitations, rigor and consistency was ensured throughout the process by adhering to JBI methodology, which provided a structured framework for conducting the systematic reviews.^31,32^

## Conclusion

HDL2 presents with highly variable clinical features, which can lead to delayed diagnosis or misdiagnosis. Healthcare professionals should consider HDL2 in patients with symptoms resembling HD, especially for patients who report having African ancestry. This systematic review highlights the need for improved understanding and awareness of early psychiatric manifestations in HDL2 patients. Longitudinal prospective study designs to ascertain motor and non-motor symptoms during the early stages of HDL2 will be informative.^88^ Several further gaps in current literature are notable, including data on repeat lengths and anticipation.^34,49,50^ Studies including individuals with *JPH3* CAG repeat sizes in the intermediate or reduced penetrance range will help to understand the associated variability and uncertainty, in turn enabling more accurate genetic counselling. Advances in understanding genetic modifiers of variability in HD could be similarly considered for HDL2, such as repeat sequence interruptions, non-coding variants and drivers of somatic expansion.^89^ Lastly, in-depth qualitative and quantitative insights into the experiences and needs of patients with HDL2 and their families is currently lacking and will be very important in providing a foundation for further research and improvements in health care outcomes. In the meantime, HD genetic testing protocols or those for similar late-onset neurodegenerative conditions can be applied for HDL2.^80^ Testing and refining these protocols with consumer involvement in the design, conduct and analysis of the research studies will help ensure they meet the specific needs of HDL2 patients and are culturally appropriate.^90^ Much of the literature on HD counselling protocols likely focuses on predominantly white European populations and may not adequately consider cultural differences within diverse populations who have African ancestry.^80^ These differences may include health beliefs, family dynamics, communication styles and decision-making processes.^90^ Hence, adapting protocols to accommodate diverse cultural perspectives can enhance the effectiveness and accessibility of genetic counselling services for individuals and families affected by HDL2.^90^ Consumer-led and/or co-designed culturally appropriate studies, including qualitative methodology to better understand needs, experiences, facilitators, barriers, and patient-reported outcomes will be critical.

## Supplemental Material

sj-docx-1-hun-10.1177_18796397251411109 - Supplemental material for Current knowledge of Huntington's disease-like 2 genetic testing, clinical presentation, and patient experiences: A systematic review Supplemental material, sj-docx-1-hun-10.1177_18796397251411109 for Current knowledge of Huntington's disease-like 2 genetic testing, clinical presentation, and patient experiences: A systematic review by Katharina Hoffmann, Stephanie White and Adrienne Sexton in Journal of Huntington's Disease
