# Supplementary material for: Current knowledge of Huntington's disease-like 2 genetic testing, clinical presentation, and patient experiences: A systematic review
Source: J Huntingtons Dis. 2026 Jan 21;15(3):293–316. doi: 10.1177/18796397251411109 (PMC13396421; doi:10.1177/18796397251411109)
Supplement: sj-docx-1-hun-10.1177_18796397251411109 - Supplemental material for Current knowledge of Huntington's disease-like 2 genetic testing, clinical presentation, and patient experiences: A systematic review [file sj-docx-1-hun-10.1177_18796397251411109.docx]

**Supplementary Material**

**Table 1:** Key words, search terms and Boolean operators used in database searches for research questions 1 and 2

| **Research Question 1: What information about HDL2 is currently available and can be used to enhance the process of genetic testing, diagnosis, and genetic counselling for HDL2?** | | | | | | | |
| --- | --- | --- | --- | --- | --- | --- | --- |
| Key words | HDL2 |  | Genetic testing |  | Diagnosis |  | Genetic counselling |
| Search terms | HDL2 NOT lipoprotein NOT cholesterol  OR “Huntington* disease-like 2” NOT lipoprotein NOT cholesterol OR “Huntington* disease like-2” NOT lipoprotein NOT cholesterol OR “Huntington* disease-like phenotype*” NOT lipoprotein NOT cholesterol OR “Huntington* disease-like” NOT lipoprotein NOT cholesterol OR “Huntington* disease-like syndrome*” NOT lipoprotein NOT cholesterol OR “Huntington-like phenotype*” NOT lipoprotein NOT cholesterol  OR “Huntington-like disorder*” NOT lipoprotein NOT cholesterol OR “Huntington* disease phenocop*” NOT lipoprotein NOT cholesterol | AND | “Gen* test*” OR  “Gen* carrier screen*” | AND | Diagnos* | AND | “Gen* counsel*” |
| **Research Question 2: What information about patient and family members experiences is currently available to enhance our understanding of what patients with HDL2 require for additional support?** | | | | | | | |
| Key words | HDL2 |  | Patient |  | Family | | |
| Search terms | HDL2 NOT lipoprotein NOT cholesterol OR “Huntington* disease-like 2” NOT lipoprotein NOT cholesterol OR “Huntington* disease like-2” NOT lipoprotein NOT cholesterol OR “Huntington* disease-like phenotype*” NOT lipoprotein NOT cholesterol OR “Huntington* disease-like” NOT lipoprotein NOT cholesterol OR “Huntington* disease-like syndrome*” NOT lipoprotein NOT cholesterol OR “Huntington-like phenotype*” NOT lipoprotein NOT cholesterol OR “Huntington-like disorder*” NOT lipoprotein NOT cholesterol OR “Huntington* disease phenocop*” NOT lipoprotein NOT cholesterol | AND | Patient* | AND | Famil* | | |

**Table 2:** Published cases of Huntington disease-like 2, adapted from Krause et al. 2024, [1] showing authors and year of the relevant publications in brackets.

| **Country of origin** | **Number of cases documented in the previous review by Krause et al. 2024**[1] | **Number of cases in this current review** |
| --- | --- | --- |
| South Africa | 40[9,10,46,51,52,61] | 44[9,10,35,40,43,46-48,51,52,55,61] |
| USA | 24[11,14,44,97-99] | 24[11,14,44,45,49,57] |
| Brazil | 12[41,42,58,59,100] | 13[38,41,42,58,59,100] |
| Venezuela | 4[50] | 11[34,50] |
| Mexico | 3[11] | 5[11,37] |
| Caribbean | 3[45,53,95] | 3 [45,53,95] |
| UK | 2[54,60] | 2[54,60,94] |
| Italy | 0 | 1[39] |
| Botswana | 1[12] | 1[12] |
| Democratic Republic of the Congo | 1[95] | 1[95] |
| Morocco | 1[101] | 1[101] |
| Mali | 0 | 3[36] |
| Total | 91 | 109 |
